# Screening of Colistin-Resistant Bacteria in Domestic Pets from France

**DOI:** 10.3390/ani12050633

**Published:** 2022-03-02

**Authors:** Afaf Hamame, Bernard Davoust, Jean-Marc Rolain, Seydina M. Diene

**Affiliations:** 1 MEPHI, IRD, APHM, IHU-Méditerranée Infection, Faculté de Pharmacie, Aix Marseille Université, 13005 Marseille, France; afafhamame@gmail.com (A.H.); jean-marc.rolain@univ-amu.fr (J.-M.R.); 2 IHU-Méditerranée Infection, 19-21 Boulevard Jean Moulin, CEDEX 05, 13385 Marseille, France; bernard.davoust@gmail.com

**Keywords:** colistin resistance, *mcr* genes, dogs, cats, pets, France

## Abstract

**Simple Summary:**

Zoonotic transmission from pets to their owners is a major health problem, especially when dealing with human pathogens. It is important to determine the reservoir of colistin-resistant bacteria in pets to avoid the risk factors for human transmission. This study investigated the screening of colistin-resistant bacteria in pets in Marseille, France. Overall, cats and dogs have various reservoirs of colistin-resistant bacteria, including naturally colistin-resistant bacteria and *mcr* gene carriers (*n* = 14). Pets are the best human companions; therefore, vigilance would be required to avoid zoonotic transmission of colistin-resistant bacteria. Although colistin use is restricted in France, we report here for the first time that cats and dogs have various colistin-resistant bacteria including *mcr*-*1* gene carriers.

**Abstract:**

Background: Pets are the closest animals to humans with a considerable risk of zoonotic transmission. This study aimed to screen colistin-resistant bacteria from stools of dogs and cats from Marseille, France. Screening of *mcr* genes in pets has never been reported in France. Methods: Fecal samples (*n* = 157) were cultivated on the selective Lucie-Bardet Jean-Marc-Rolain medium (LBJMR). Bacteria were identified using Microflex LS MALDI-TOF. The antibiotic resistance phenotype was investigated for several antibiotics (β-lactams, aminoside, cephalosporine, tetracycline, and sulfonamide). PCR techniques were performed to detect *mcr* genes. Results: A total of 218 bacteria were identified. For cats, intrinsically colistin-resistant bacteria were significantly higher than *mcr-1* gene carriers (*n* = 4). Dogs had more bacteria with the *mcr-1* gene (*n* = 10). Furthermore, cats had a high prevalence of Gram-positive bacteria (GPB), whereas dogs had GNB equal to GPB. The diversity of identified bacteria was due to the constitution of the pets’ microorganisms. Even though colistin use is monitored in France, pets harbor various colistin-resistant bacteria. Additionally, in this geographical area, bacteria bearing *mcr-1* gene from dogs and cats were detected for the first time. Conclusions: The current study opens a new perspective: the spread of colistin resistance is independent of colistin use. What are the most factors related to the emergence of colistin resistance? The surveillance of pets must be considered a priority to avoid the spread of *mcr* genes. It is important to know the contribution that pets make to the pool of multidrug-resistant *mcr-1*-containing bacteria.

## 1. Introduction

Colistin (polymyxin E) has been used as a growth promoter in food-production animals, but it is also used in pets for the prevention and treatment of bacterial infections. As previously reported, the overuse of colistin has been shown to cause colistin resistance in bacteria colonizing the intestinal gut of animals [1]. However, only a few countries have prohibited the long-term use of colistin. Colistin reuse has resurfaced due to necessity and even more limited treatment options, particularly since the spread of multidrug-resistant bacteria (MDR) and carbapenem-resistant bacteria [2]. Colistin-resistant bacteria have become a significant public health issue. Indeed, colistin is a last-resort antibiotic; its failure to treat patients necessitates the development of new and more effective antibiotic therapies [3]. Since the discovery of the first plasmid harboring *mcr-1* in China from pigs, the microbiota of animals appears as a source of colistin-resistant bacteria [4]. A variety of colistin-resistance gene variants have been discovered, ranging from *mcr-2* to *mcr-10* [5,6,7,8,9,10,11,12,13]. In 2016, *mcr-1* was detected in several samples from humans and from food-producing animals on all seven continents and in more than 30 countries [14]. It is crucial to have a better understanding of the microorganisms found in pets, as well as the risk of pathogen transmission and antibiotic resistance genes such as *mcr-1*. *mcr-1-*producing bacteria have been reported in zoonotic transmission from pets to humans who adopt them for protection, entertainment, or companionship [15]. Close contact between cats, dogs, and their owners led to the occurrence and transmission of antibiotic-resistant microorganisms [16].

In Asia and the United States, *mcr-1* in *Enterobacteriaceae* has already been reported in dogs and cats. The *mcr* genes in pets were also detected in Beijing, China, between 2012 and 2016 [17]. Further studies reported that dog feces in a city park in Quito (Ecuador) have a high prevalence of multidrug-resistant bacteria (MDR) [18]. Between 2017 and 2019, researchers in China reported an abundance of MDR such as *Klebsiella pneumoniae* harboring colistin resistance genes (*mcr-1*, *mcr-8*) and β-lactamases (*bla*_OXA-181_, *bla*_NDM-5_) in cats, which was significantly higher than in dogs [19]. In Europe, almost all colistin resistance studies have been focused in food-producing animals such as pigs and poultry [20,21,22]. Regarding Asia, for the first time, the *mcr-1* gene was detected in dogs from South Korea, with an average nucleotide identity analysis similar to those found in Korean patients [23]. However, diseased dogs from Taiwan have *Klebsiella spp* and *Enterobacter spp* carrying *mcr-1* gene [24].

Interestingly, colistin-resistant strains in pets have never been investigated in France, according to the literature. Colistin is currently not recommended for treating animal infections caused by *Enterobacteriaceae* in France unless it is necessary. Colistin is a critically important antibiotic that is used as a last resort treatment. Furthermore, because of unfavorable and toxic effects of colistin, its restriction has been requested by some agricultural or veterinary organizations and also by the European commission in 2016 [1]. Despite these precautions, the emergence of colistin resistance in animals is becoming more common, as evidenced by various studies on the subject. As reported, colistin resistance in animals is likely mediated by environmental factors and animal nutrition [25].

Colistin resistance in pets is rarely studied because the majority of reported studies were focused on food animals. However, pets are in daily close contact with humans. Determining the prevalence of colistin-resistant bacteria in pets is critical in order to identify any potential risk factors for colistin resistance transmission, particularly zoonotic transmission of bacteria [26].

In the current study, in a One Health approach, we aimed to investigate colistin-resistant bacteria in dogs and cats. We also reported on the screening of *mcr* genes that can greatly contribute to a large diffusion of colistin resistance in pets but also in humans.

## 2. Materials and Methods

### 2.1. Sample Collection

In France, fecal samples were taken from pets between 2019 and 2020. This study included 157 samples from pets’ feces (52 dogs and 105 cats). The cats and dogs were living in shelters in Marseille, Bouches-du Rhône department of France. Adult dogs and cats ranging in age from 1 to 8 years were used for the current study. Among the dogs, 60% males and 40% were females; among the cats, half were males and half females. All of the animals had been spayed or neutered. The dogs and cats were in good health, and no antibiotic therapy had been used. The dogs were in boxes with outdoor kennels and the cats were in enclosures.

The fecal collection was performed with veterinarian intervention to obtain fresh stools. One fecal sample per animal was collected rectally using a sterile cotton swab and wooden medical spatula. All collected samples were stored at −80 °C until processing. It is important to note that these animals were in daily contact with customers, buyers, and pet lovers.

### 2.2. Ethics Statement

The IHU Mediterranee Infection is authorized to use animal samples in categories one, two, and three for diagnostic purposes and research. Authorization was granted by the prefecture (Bouches-du-Rhône) under N° 13-205-107 on 4 September 2014. The decree of 8 December 2011 lays down health rules concerning animal by-products and derived products in the application of regulation (EC) N° 1069/2009 and regulation (EU) N° 142/2011.

### 2.3. Screening for Colistin-Resistant Bacteria

For bacterial enrichment, all samples were cultured in TSB, and enrichment samples were cultured on LBJMR medium containing vancomycin (50 g/mL) and colistin (4 g/mL). Growing bacteria in LBJRM were selected according to their size, shape, and color. As already known, *Enterobacteriaceae* can appear differently in LBJMR with colony sizes ranging from 2 to 3 mm. *Enterococci* colonies were very small (0.1–1 mm) [27].

### 2.4. Bacterial Identification

Different colonies representative of each morphological category were picked up from LBJMR agar plate and then identified using matrix-assisted laser desorption/ionization time-of-flight mass spectrometry with a Microflex LS MALDI-TOF spectrometer (Bruker Daltonics, Bremen, Germany). Protein profile spectra were compared to those of databases harboring several bacterial spectra: Culturomics, BDAL, and Timone. Identification was validated only when the score was between 1.8 and 2.0. Bacteria that were difficult to identify with low scores because of their fatty texture were exposed to protein extraction to obtain better scores.

### 2.5. Antibiotic Susceptibility Tests (AST)

Using the Kirby–Bauer disk diffusion method, AST was performed to explore antibiotic resistance phenotypes. All the non-naturally colistin-resistant bacteria isolated from the LBJMR medium were tested using antimicrobial susceptibility tests, according to the current disk-diffusion test method (Kirby–Bauer procedure). Antibiotic disks diffuse a specific concentration of antibiotics. The bacterial suspension in NaCl must have definite turbidity (0.5 McFarland). Sixteen antimicrobial agents were used including Amoxicillin (AMX), Amoxicillin-clavulanic acid (AMC), Cefepime (FEP), Piperacillin/Tazobactam (TPZ), Cefalotin (KF), Ceftriaxone (CRO), Ertapenem (ETP), Imipenem (IMP), Fosfomycin (FF), Nitrofurantoin (F), Trimethoprim-sulfamethoxazole (SXT), Amikacin (AK), Ciprofloxacin (CIP), Doxycycline (DO), Colistin (CT), and Gentamicin (GN) (Bio-Rad, Marne-la-Coquette, France). The antibiotic disk diffused into the agar plate, preventing bacterial growth, and this appeared in the transparent zone around the disk that is called the inhibition zone.

In addition, isolated bacteria with a narrow diameter zone of inhibition (ZOI) around the colistin disk of less than 14 mm were subjected to further tests to confirm the colistin resistance phenotype. The minimal inhibition concentration (MIC) was identified using the E-test method (BioMerieux) and UMIC (Biocentric Bandol, France). Strains were considered resistant or sensitive to colistin according to reference minimal inhibition concentration (MIC) values listed in EUCAST guidelines [28]. Here, the colistin break-point was ≥2 μg/mL. However, strains were considered MDR if they were resistant to more than three different classes of antibiotics. Hierarchical clustering of the antibiotic resistance phenotype was performed using Multi-Experiment Viewer (MeV 4.9.0).

### 2.6. DNA Extraction

DNA extraction was performed using the EZ1 DNeasy Blood Tissue Kit (Qiagen GmbH, Hilden, Germany). The absorbance readings referring to DNA purity were between 260 and 280 nm (Spectrophotometer ND-100, Nanodrop Thermo Fisher Scientific, Wilmington, DE, USA).

### 2.7. Molecular Characterization of Colistin Resistance Genes

Several bio-molecular methods, real-time reaction PCR (qPCR), standard PCR (ST-PCR), and Sanger sequencing, were performed to screen colistin resistance genes in bacteria with MIC_col_ ≥ 2µg/mL. All of the GNB including naturally colistin-resistant strains were subjected to the *mcr* gene screening tests because it had already been reported that *Proteus mirabilis* harbor the *mcr* gene [29]. As *mcr* can be found in naturally colistin-resistant bacteria, PCR tests were also performed for GPB. The choice of *mcr* variant was based on the fact that *mcr* genes are already found in animals. The colistin resistance genes tested in this study were the following: *mcr-1*, *mcr-2*, *mcr-3*, *mcr-4*, *mcr-5*, *mcr-8*. The screening of *mcr* genes was investigated by qPCR using CFX96 TM Real-time system/C and ST-PCR. Those methods were performed using specific primers developed in our laboratory [30]. Supplementary Table S1 shows primers used for this study to screen *mcr* genes. The RT-PCR assay conditions were as follows: 50 °C for 2 min; 95 °C for 15 min; 95 °C for 1 s; 60 °C for 30 s ×30 cycles; 45 °C for 30 s. The sets were confirmed with negative and positive controls (*Escherichia coli* and *K. pneumoniae*) harboring plasmids with *mcr* genes and *E. coli ATCC 25*,*922* for the negative control. We considered that bacteria carried *mcr* gene when qPCR reactions resulted in a cycle threshold value inferior to 35. Further biomolecular tests were performed to confirm the positive pattern against the target gene (ST-PCR and Sanger sequencing). All results were confirmed by Sanger sequencing and by the blast of *mcr* gene sequence, followed by alignment.

### 2.8. Statistical Analysis

The accumulated data, including identification and prevalence, were represented in the respective animal population as relative frequency (percentage).

## 3. Results and Discussion

### 3.1. Screening of Colistin-Resistant Bacteria in Pets

**Figure 1:**
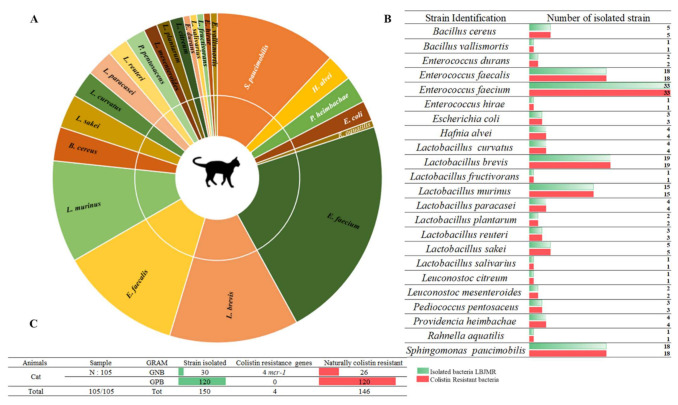
Proportion of colistin-resistant bacteria in feces of cats. (**A**). Pie chart representing the distribution of strains isolated in LBJMR. (**B**). Colistin-resistant bacteria verified by AST testing. (**C**). Statistical data on isolated bacteria according to Gram status and resistance mechanism.

Among 157 collected fecal samples from domestic animals (dogs and cats), a total of 218 bacterial isolates were obtained from selective medium agar LBJMR (Vancomycin and colistin). As already reported, the gastrointestinal tract of animals can harbor a highly complex mixture of organisms that help maintain intestinal flora and overall health [31]. Indeed, the study of fecal samples depends upon the natural composition of the microorganisms and is specific to each animal. According to the literature, most studies have been performed on the general microorganism composition and most data have been derived from the analysis of feces from healthy laboratory animals [32,33]. In our study, the composition of microorganisms in the feces samples was described in terms of naturally colistin-resistant bacteria and those with acquired colistin resistance via *mcr* genes. Identification of colistin-resistant bacteria in cats revealed 150 bacteria, as represented in Figure 1. Identification encompassed 23 different bacterial species. Eighty percent (*n* = 120) of all identified bacteria were Gram-positive and 20% (*n* = 30) were Gram-negative. The dominant strains were naturally colistin-resistant for all GPB: *Bacillus cereus, B. vallismortis, Enterococcus durans*, *E. faecalis*, *E. faecium*, *E. hirae*, *Leuconostoc citreum*, *L. mesenteroides*, and *Pediococcus pentosacens*. A high proportion of *Lactobacillus* species were identified: *L. curvatus, L. brevis*, *L. fructivorans*, *L. murinus*, *L. paracasei*, *L. planetarium, L. reuteri*, *L. sakei*, and *L. salivarius*. In contrast, the GNB that were isolated were believed to have acquired resistance to colistin via *mcr* genes or other mechanisms. Here, 13% (*n* = 4) of colistin-resistant GNB were screened as being *mcr* carriers: *E. coli* (*n* = 3) and *Rahnella aquatilis* (*n* = 1). In addition, 87% (*n* = 26) of GNB strains were naturally resistant to colistin: *Sphingomonas paucimobilis*, *Providencia heimbachae*, and *Hafnia alvei* (Figure 1). 

**Figure 2:**
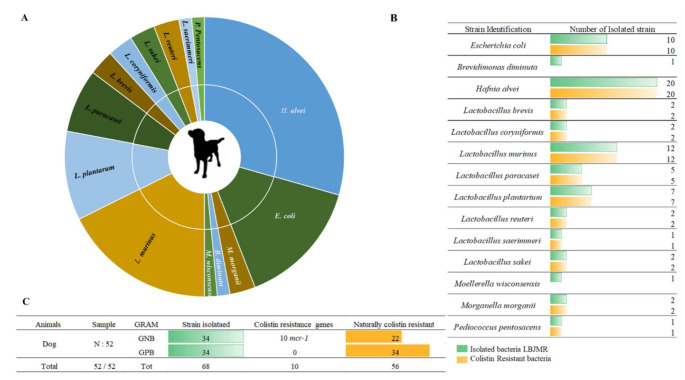
Illustration of colistin-resistant bacteria in the feces of dogs. (**A**). Distributed representation of colistin-resistant bacteria in a pie chart. (**B**). Distinguished colistin-resistant bacteria isolated in LBJMR. (**C**). Statistical analysis according to Gram status and colistin resistance.

The selective bacterial culture of dogs’ feces allowed us to isolate a total of 68 bacterial strains, divided into 14 different species. Fifty percent (*n* = 34) of the isolated strains were Gram-negative and 50% (*n* = 34) were Gram-positive. The isolated GPB in dogs’ feces were: *L. brevis*, *L. coryniformis*, *L. murinus*, *L. paracasei*, *L. planetarium*, *L. reuteri*, *L. saerimmeri*, *L. sakei* and *P. pentosacens*. The Gram-negative strains corresponded to *E. coli*, *Moellerella wisconsensis* and *Brevundimonas diminuta*. Sixty-five percent (*n* = 22) of the GNB were naturally resistant to colistin: *H. alvei* and *Morganella morganii* (Figure 2). Colistin-resistant bacteria differ widely from one animal to another. As mentioned previously, bacteria in fecal samples from dogs exhibited broad variability [34]. Generally, colistin is used orally, with low bioavailability, so the gastrointestinal microorganisms are directly exposed to colistin [35]. In 2016, the polymyxin family was the fifth best-selling antibiotic in Europe, and among polymyxins, colistin accounted for more than 99.9% of sales [36]. This uncontrollable use accelerated the dissemination of colistin resistance in animals and, subsequently, human beings [37]. Consequently, in 2016 the European Medicines Agency (EMA) updated its guidelines to minimize the use of colistin in animals to reduce its impact on human health [36,38]. The restricted colistin use in cats and dogs in France has not prevented the abundance of colistin-resistant bacteria in their microbiome composition. These findings highlight a topic that requires more research on the sources of colistin resistance.

**Figure 3:**
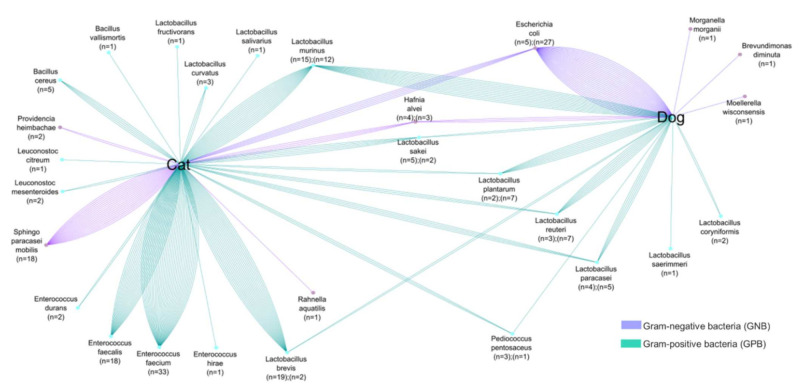
Comparative analyses of common colistin-resistant bacterial species found in the fecal samples of dogs and cats. The number of strains is proportional to the number of lines.

Cats and dogs, on the other hand, share certain bacterial species; however, as shown in Figure 3, colistin-resistant bacteria in cat and dog feces are variable. In cats, we isolated a variety of species of the genera: *Lactobacillus*, *Bacillus*, and *Enterococcus*, whereas in dogs *Lactobacillus* was the most abundant genus, with seven different species. Moreover, GNB isolated in cats and dogs are different and do not belong to the same bacterial genera. In addition, cats and dogs also share certain bacterial species. The bacterial cross-link between cats and dogs corresponds to the following bacteria: GNB (*E. coli*, *H. alvei*, *P. heimbachae* and *S. paracaseimobilis*); and GPB (*L. murinus*, *L. plantarum*, *L. sakei*, *L. reuteri*, *L. paracasei*, *L. brevis* and *P. pentosaceus*). In colistin-resistant bacteria, natural colistin resistance is stronger than acquired resistance. It is interesting to note that both humans and animals can have a cross-link of identical bacterial species in their stools, which could lead to bacterial exchanges between the two species [39].

### 3.2. Phenotype of Antibiotic Resistance

The minimal inhibition concentration of the antibiotic was attributed to the strain according to 2017 European Committee on Antimicrobial Susceptibility Testing (EUCAST) guidelines. Almost all of the isolated strains were colistin-resistant, with an inhibition zone of less than 15 mm. It is important to note that AST tests in agar plate (E-test and disk-diffusion test method) are limited to the screening of colistin resistance and may have missed some isolates [40]. Regarding the carbapenem family, only 5% of those species, *E. coli*, *M. morganii* and *B. diminuta*, were resistant to carbapenem. The same proportion held for imipenem: *E. coli*, *S. paucimobilis* and *M. morganii*. In addition, 21% of bacteria were resistant to amoxicillin and amoxicillin clavulanic acid: *P. heimbachae*, *S. marcescens* and *E. coli*. All of the *S. paucimobilis* isolated in cats was resistant to piperacillin/tazobactam (penicillin class), as well as (*n* = 1) *E. coli* from dogs’ feces. Twenty-two percent of the strains were resistant to cephalosporin antibiotics (cefalotin and ceftriaxone). This phenotypic resistance concerned *S. paucimobilis*, *E. coli*, *M. morganii* and *B. diminuta* species. Antibiotic resistance phenotype is illustrated in Supplementary Materials Figures S1 and S2. The presence of MDR bacteria in pets suggests a potential sharing of resistant bacteria between pets and humans. It has been proved that dogs present high levels of MDR against the large β-lactams family. Antibiotic resistance is often linked to the overuse and misuse of antibiotics such as sulfonamides, trimethoprim, and clindamycin, which are commonly used in animals [41].

### 3.3. Screening of Mobile Colistin Resistance (mcr) Genes in Fecal Samples from Pets

This study revealed the existence of *mcr-1* in cats and dogs for the first time in France (Table 1). In cats, *E. coli* (*n* = 3) and *Rahnella aquatilis* (*n* = 1) were screened as *mcr-1* carriers (CT < 34) and had a minimum inhibitory concentration (MIC) for colistin of 8 µg/mL–64 µg/mL. Regarding the dog samples (*n* = 10), *E. coli* harbored *mcr-1* (CT < 35) and had an MIC range for colistin between 4 µg/mL and 12 µg/mL. Sanger sequencing and BLAST results demonstrated the presence of *mcr-1* gene with more than 96% identity and coverage of not less than 93%. The fact that such a large number of *mcr-1* genes were found in dogs raises the possibility that these genes are transmissible via recombinant plasmid carrying *mcr-1*. 

Bacteria carrying *mcr* genes in dog fecal samples are not very common. *mcr* genes were detected in *E. coli* and *Pseudomonas aeruginosa* [41]. However, dogs and their owners have high potential colonization of antibiotic-resistant bacteria [42]. Colistin-resistance genes have also recently been detected in dogs in South Korea [23]. Further studies have assessed the occurrence of *mcr-1* and *mcr-2* producing *Enterobacteriaceae* in cats and other companion animals in Switzerland as well as other countries [24,43,44,45,46,47]. For the first time in France, we reported colistin-resistant bacteria carrying *mcr* genes in pets, although we cannot state if colistin resistance comes from contact with humans, food contamination, illegal colistin use, or environmental factors. It is important to know that colistin use is limited in France and especially in domestic animals to avoid the development of resistance in these animals despite the possibility that this resistance may not be directly associated with colistin use. So far, colistin use represents 6% of antibiotic use in pets, and it is classified by the World Health Organization (WHO) as a critically important antimicrobial of highest priority [46].

This study highlights the antibiotic resistance concern, in a One Health approach. Human beings share their daily lives with pets that provide psychological support, faithful companionship, and enjoyment. The *mcr* gene found in pets can be easily transmitted to humans. Zoonotic transmission from pets to their owners has already been reported in several studies. It is important to establish strict hygiene conditions and diverse approaches to reduce zoonotic transmission of *mcr*-producing bacteria [42,47,48]. Bacteria that carry *mcr* genes can cause diseases such as urinary tract infections, previously reported to be linked to the presence of *E. coli* carrying the *mcr-1* gene [47]. Pneumonia and respiratory diseases have been diagnosed in pets and their owners and the pneumopathy was associated with the presence of colistin-resistant strains [48].

## 4. Conclusions

The colistin-resistant bacteria present in the fecal samples of cats and dogs appear highly diverse. Many factors may be involved in the emergence of colistin resistance, including illegal colistin use and *mcr* genes that have a high potential for transfer. In order to track the emergence of *mcr* genes in domestic animals in France, prospective studies will be carried out in other locations. Greater vigilance is required to avoid zoonotic transmission, which may represent the infection route for humans and pets via colistin-resistant bacteria because of their close contact. However, these pets may be contaminated also by resistant bacteria from the environment or nutrition products. Adopters of puppies and kittens must establish strict hygiene conditions in their homes to prevent the spread of antibiotic resistance genes.

## Figures and Tables

**Table 1:** Biomolecular results and colistin MIC of resistant bacteria harboring *mcr* genes isolated from dogs and cats.

Geographical Location	Animal	Strain	Colistin Resistance Gene	q-PCR (Ct)	St-PCR	MIC UMIC (µg/mL)	Identity (%)	E-Value
Provence Alpes-Côte D’Azur (Marseille)	Cat	*E. coli*	*mcr-1*	34	+	8	99	0.0
		*E. coli*	*mcr-1*	32	+	4	98	0.0
		*E. coli*	*mcr-1*	31	+	2	99.1	0.0
		*Rahnella aquatilis*	*mcr-1*	29	+	64	97.2	0.0
	Dog	*E. coli*	*mcr-1*	34	+	4	99.4	0.0
		*E. coli*	*mcr-1*	35	+	4	99.1	0.0
		*E. coli*	*mcr-1*	30	+	2	96	0.0
		*E. coli*	*mcr-1*	34	+	12	99	0.0
		*E. coli*	*mcr-1*	30	+	4	99.4	0.0
		*E. coli*	*mcr-1*	25	+	8	97.8	0.0
		*E. coli*	*mcr-1*	21	+	6	98	0.0
		*E. coli*	*mcr-1*	29	+	2	99.4	0.0
		*E. coli*	*mcr-1*	30	+	8	99.3	0.0
		*E. coli*	*mcr-1*	29	+	2	98.5	0.0

## Data Availability

Not applicable.

## Supplementary Materials

The following supporting information can be downloaded at: https://www.mdpi.com/article/10.3390/ani12050633/s1, Supplementary Table S1: Sequences of primers and probes used for real-time PCR and conventional PCR assays in this study. Supplementary Figure S1: Hierarchical clustering of antibiotic resistance phenotype of bacteria using Multi-Experiment Viewer (MeV 4.9.0). Antibiotic resistance pattern of bacteria isolated from cats. Supplementary Figure S2: Hierarchical clustering of antibiotic resistance phenotype of bacteria using Multi-Experiment Viewer (MeV 4.9.0). Antibiotic resistance pattern of bacteria isolated from dogs.
