# Supplementary material for: Screening of Colistin-Resistant Bacteria in Domestic Pets from France
Source: Animals (Basel). 2022 Mar 2;12(5):633. doi: 10.3390/ani12050633 (PMC8909117; doi:10.3390/ani12050633)
Supplement: Supplementary file 1 [file animals-12-00633-s001.zip › animals-1568269 -supplementary/Table S1.pdf]

**Supplementary Table S1**: Sequences of primers and probes used for real-time PCRs and conventional PCRs in this study.

| Gene | PCR Type | Primers/Probes | PCR Product (bp) |
|---|---|---|---|
| *mcr*-1 | RT-PCR | F: GCAGCATACTTCTGTGTGGTAC<br>R: ACAAAGCCGAGATTGTCCGCG<br>Probe: FAM-GACCGCGACCGCCAATCTTACC-TAMRA | **180** |
| | Standard PCR and sequencing | F: GCAGCATACTTCTGTGTGGTAC<br>R: TATGCACGCGAAAGAAACTGGC | **893** |
| *mcr*-2 | RT-PCR | F: CTGTGCCGTGTATGTTCAGC<br>R: TTATCCATCACGCCTTTTGAG<br>Probe: VIC-TGACCGCTTGGGTGTGGGTA-TAMRA | **151** |
| *mcr*-3 | RT-PCR | F: TGAATCACTGGGAGCATTAGGGC<br>R: TGCTGCAAACACGCCATATCAAC<br>Probe: FAM-TGCACCGGATGATCAGACCCGT-TAMRA | **144** |
| | Standard PCR and sequencing | F: AAATAAAAATTGTTCCGCTTATG<br>R: ATGGAGATCCCCGTTTTT | **556** |
| *mcr*-4 | RT-PCR | F: GCCAACCAATGCTCATACCCAAAA<br>R: CCGCCCCATTCGTGAAAACATAC<br>Probe: FAM-GCCACGGCGGTGTCTCTACCC-TAMRA | **112** |
| *mcr*-5 | RT-PCR | F: TATCCCGCAAGCTACCGACGC<br>R: ACGGGCAAGCACATGATCGGT<br>Probe: FAM-TGCGACACCACCGATCTGGCCA-TAMRA | **126** |
| *mcr*-8 | RT-PCR | F: TCCGGGATGCGTGACGTTGC<br>R: TGCTGCGCGAAT-GAAGACG<br>Probe: FAM-TCATGGAGAATCGCTGGGG-GAAAGC-TAMRA | **158** |
