# Supplementary figures and images for: Screening of Colistin-Resistant Bacteria in Domestic Pets from France
Source: Animals (Basel). 2022 Mar 2;12(5):633. doi: 10.3390/ani12050633 (PMC8909117; doi:10.3390/ani12050633)

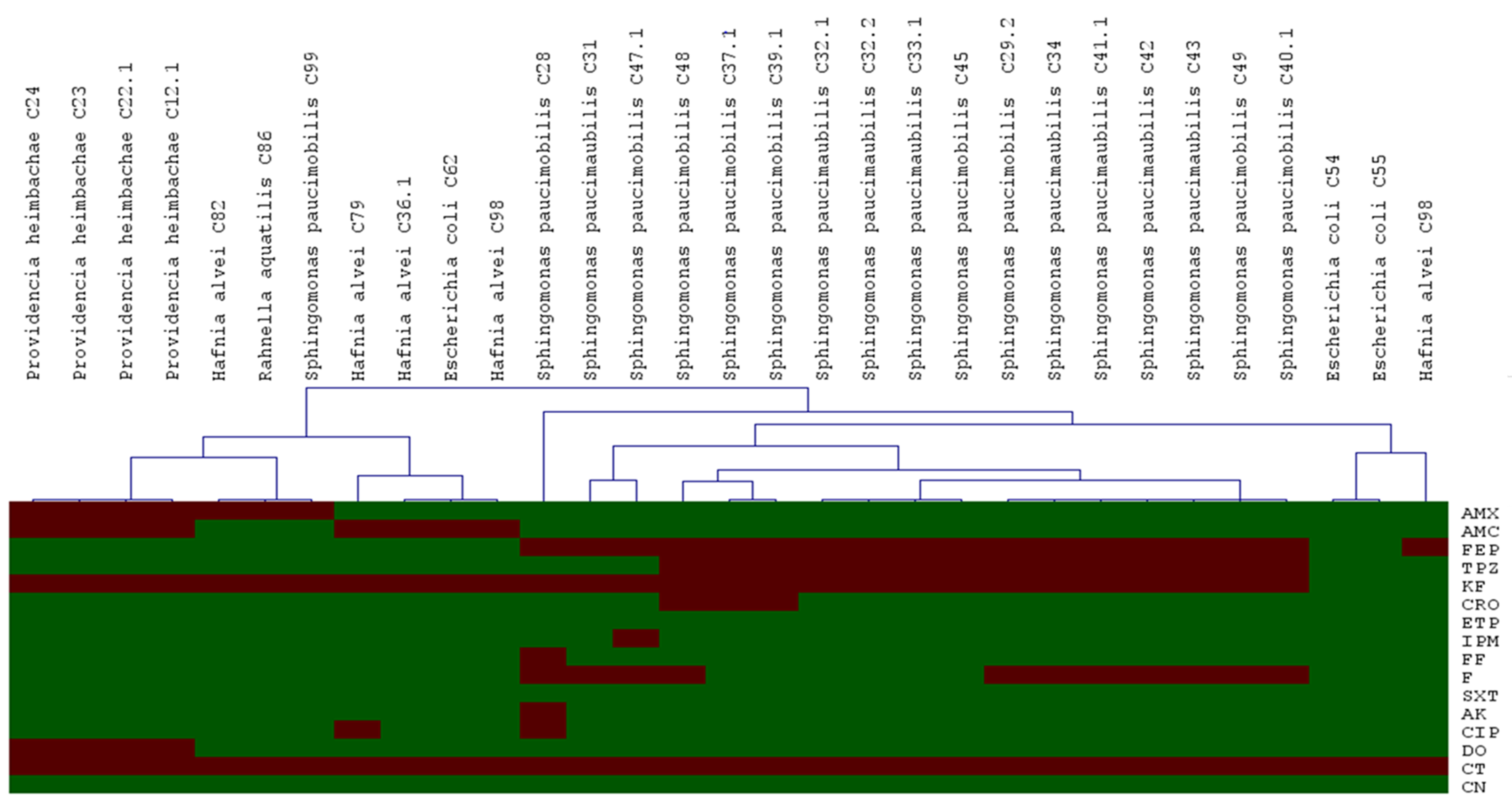

Supplement: Supplementary file 1 [file animals-12-00633-s001.zip › animals-1568269 -supplementary/Figure S1 Hierarchical clustering of antibiotic resistance phenotype of bacteria using Multi-Experiment Viewer (MeV 4.9.0). Antibiotic resistance pattern of bact.tif]

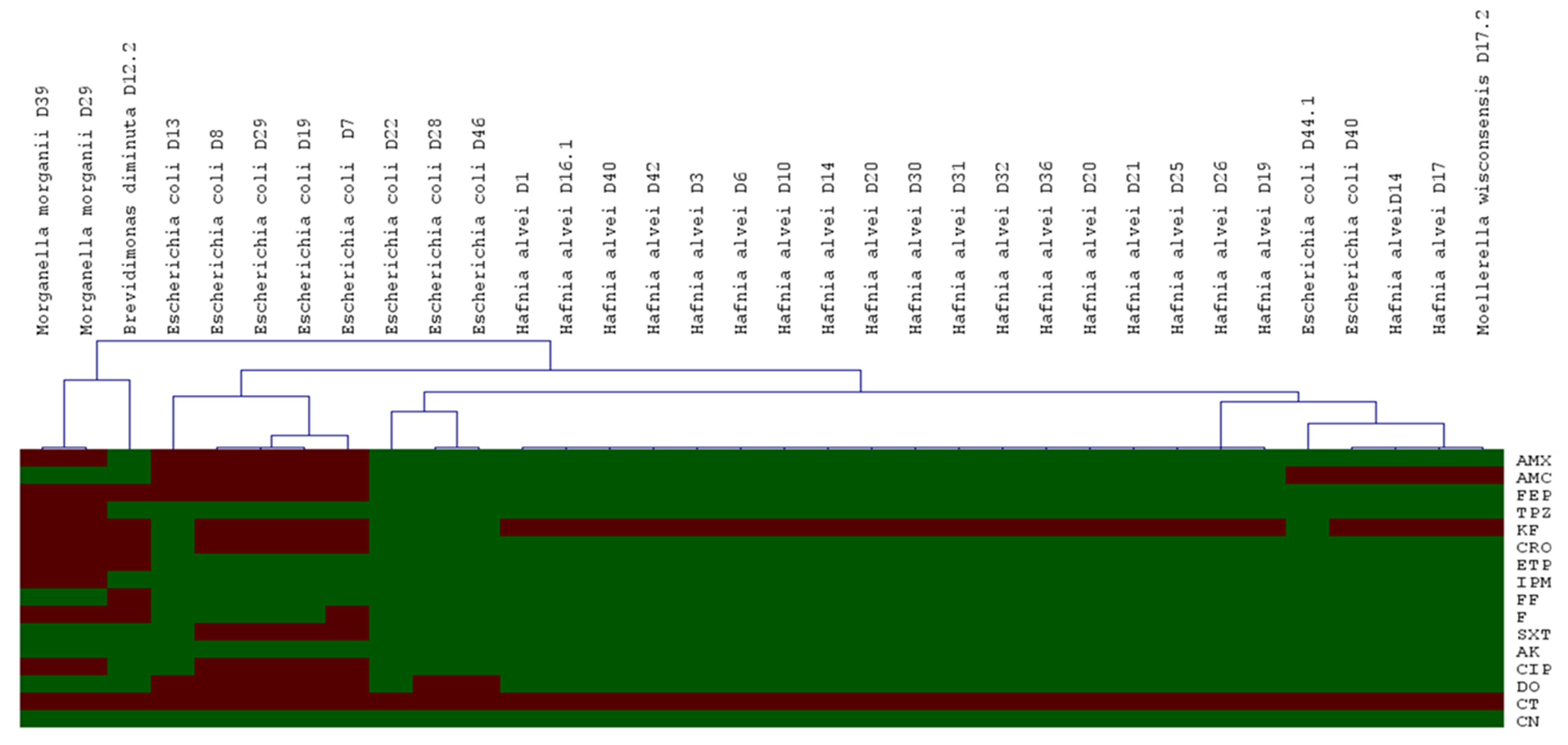

Supplement: Supplementary file 1 [file animals-12-00633-s001.zip › animals-1568269 -supplementary/Figure S2 Hierarchical clustering of antibiotic resistance phenotype of bacteria using Multi-Experiment Viewer (MeV 4.9.0). Antibiotic resistance pattern of bact.tif]
